# Comparison of the Morphological and Physical Properties of Different Absorbent Wound Dressings

**DOI:** 10.1155/2018/9367034

**Published:** 2018-05-21

**Authors:** Sukhontha Hasatsri, Anuphap Pitiratanaworanat, Suwit Swangwit, Chadaporn Boochakul, Chamaipond Tragoonsupachai

**Affiliations:** Department of Pharmacy Practice, Faculty of Pharmacy, Rangsit University, Pathum Thani, Thailand

## Abstract

Good quality wound dressings should have exceptional properties for usage, such as being able to remove excess wound exudates, having rapid dehydration, and providing optimal water vapour permeability. This study evaluated and compared the morphological and physical properties of six different commercially absorbent wound dressings in Thailand: two hydrocolloids, two alginates, and two foams. These wound dressings are available in a variety of components and structures, some of which have a multilayer structure. The results showed that the calcium sodium alginate dressings had better absorption properties than the calcium alginate dressings, hydrocolloid dressings, hydrocolloid with foam layer dressings, foam with polyurethane film layer dressings, and foam with hydrogel and polyurethane film layer dressings. Furthermore, the calcium sodium alginate dressings had the highest rate of dehydration and provided an optimal water vapour transmission rate. However, the calcium sodium alginate dressings could not retain the original structure after being submerged with a wound exudate.

## 1. Introduction

The selection of a wound dressing is usually based on a wound's characteristics [1, 2]. In addition, for a wound dressing to be of good quality, it needs to have specific properties including the ability to maintain a moist environment, absorb exudate, minimise maceration to the edges of the wound, permit exchanges of bodily gas, be easy to remove, and minimise pain from the wound [3, 4]. Wounds with high exudate require absorbent wound dressings with a high absorption capacity and rapid dehydration to avoid maceration [4]. In addition, wound dressings with optimal gas exchange between the exterior and interior of the dressing or an optimal water vapour transmission rate (WVTR) are able to maintain an optimal environment for the wound to heal [5]. The dispersion of a wound dressing can cause trauma during removal of the dressing because it adheres, and as nerve endings are exposed, that can be painful. Therefore, the ideal wound dressings should minimise pain and trauma to the wound [6]. Absorbent wound dressings, commonly used in wound care, can be categorized into three types: hydrocolloids, alginates, and foams. However, films and hydrogels are nonabsorbent types of dressings [4, 7, 8]. This study focused on commercially available absorbent wound dressings in Thailand that were categorized into three groups: two hydrocolloids, two alginates, and two foams in which some of them had a multilayer structure. The wound dressings were evaluated and compared* in vitro*.

## 2. Materials and Methods

### 2.1. Materials

A number of materials were used in this study. These included hydrocolloid dressing (Nexcare™) manufactured by the 3 M Company, Minnesota, United States of America; hydrocolloid with a foam layer dressing (DuoDERM®CGF™) produced by ConvaTec Inc., New Jersey, United States of America; calcium alginate dressing (Algisite®M) manufactured by Smith and Nephew Public Limited Company, London, United Kingdom; calcium sodium alginate dressing (Kaltostat®) made by ConvaTec Inc., Deeside, United Kingdom; foam with a polyurethane film layer dressing (Allevyn®) manufactured by Smith and Nephew Public Limited Company, London, United Kingdom; and foam with a hydrogel and polyurethane film layer dressing (Askina®) produced by B. Braun Hospicare Ltd., County Sligo, Ireland. Sodium chloride and calcium chloride dihydrate were analytically graded without further purification.

### 2.2. Morphological Properties

The morphology of each dressing was assessed with a scanning electron microscope (SEM, JSM-6610 LV, JEOL) with upper (50x), lower (50x), and cross-sectional (15x) images being recorded at different magnifications. The pore size of the dressing was measured using ImageJ® software and represented in a mean ± standard deviation.

### 2.3. Absorption Properties

The absorption properties of the dressing were examined using BS EN 13726-1: 2002, Part 1: the aspects of absorbency, Section 3.2: free swell absorptive capacities with slight modifications [9]. A dressing (2 cm × 2 cm) was prepared. A test solution (8.298 g of NaCl (0.142 mol/L) and 0.367 g of CaCl_2_2H_2_O (0.0025 mol/L) were added to one litre of deionised water) represented a pseudo-wound exudate. The dressing was immersed in the test solution and then incubated at 37°C. At different periods, the dressing was removed and weighed. The experiments were performed in triplicate, and the weight increase was represented in a percentage and mean ± standard deviation.

### 2.4. Dehydration Properties

The dressing (2 cm × 2 cm) was immersed in the test solution for 30 minutes. After that, the dressing was removed, weighed, and incubated in an oven at 37°C. At different periods, the dressing was weighed. The experiments were performed in triplicate, and the dehydration rate was represented in a mean ± standard deviation [10].

### 2.5. Water Vapour Transmission Rate

The water vapour transmission rate (WVTR) of the dressing was examined using BS EN 13726-2: 2002, Part 2: the moisture vapour transmission rate of permeable film dressings with slight modifications [11]. The dressing (5 cm × 5 cm) was prepared, then a bottle of the test solution was covered with the dressing. The positive control was the bottle of the test solution that had no cover, and the negative control was the bottle of the test solution covered with a paraffin film. All of them were incubated in an oven at 37°C. At different periods, they were weighed. The experiments were performed in triplicate, and the WVTR was represented in a mean ± standard deviation.

### 2.6. Dispersion Characteristics

The dispersion characteristics of the dressing were examined using BS EN 137262: 2002, Part 1: the aspects of absorbency, Section 3.6: dispersion characteristics with slight modifications [12]. The dressing (2 cm × 2 cm) was immersed in the test solution and shaken for 60 seconds at 100 revolutions per minute. After that, the integrity of the dressing was visually established. The absorbance of the collected test solution was measured by using a UV-spectrophotometer at 200–400 nm.

## 3. Results and Discussion

### 3.1. Morphological Properties

**Figure 1:**
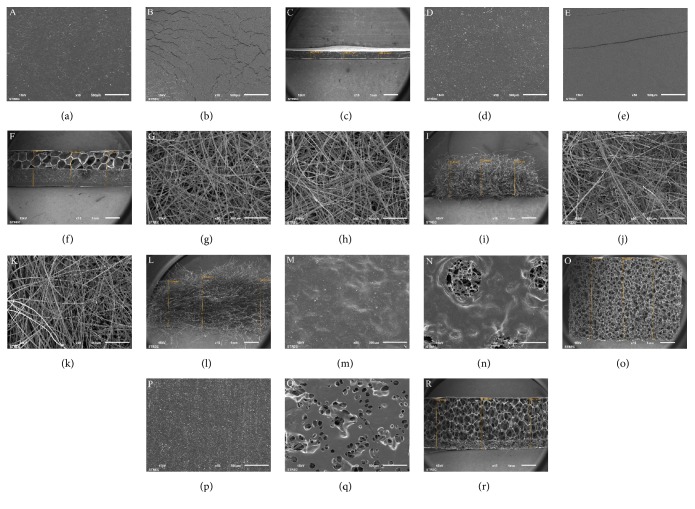
SEM photograph of (a) the upper surface, (b) lower surface, and (c) cross section of a hydrocolloid dressing; (d) the upper surface, (e) lower surface, and (f) cross section of hydrocolloid with a foam layer dressing; (g) the upper surface, (h) lower surface, and (I) cross section of a calcium alginate dressing; (j) the upper surface, (k) lower surface, and (l) cross section of a calcium sodium alginate dressing; (m) the upper surface, (n) lower surface, and (o) cross section of foam with a polyurethane film layer dressing; (p) the upper surface, (q) lower surface, and (r) cross section of foam with a hydrogel and polyurethane film layer dressing.

Upper, lower, and cross-sectional images of the wound dressings are shown in Figure 1. These results show that the thickness of the foam with a polyurethane film dressing was the highest. A wound contact surface (lower image) of foam with a hydrogel and polyurethane film layer dressing showed a greater spread pore structure than foam with a polyurethane film layer dressing. The pore size of hydrocolloid with a foam layer dressing, foam with a polyurethane film dressing, and foam with a hydrogel and polyurethane film layer dressing were 600.18 ± 95.40, 297.88 ± 26.51, and 568.42 ± 78.63 *μ*m, respectively. However, the number of pores of foam with a polyurethane film dressing was higher when compared with the other dressings. In addition, cell attachment, migration, and proliferation are an important process for the healing of a wound and an appropriate pore size range for these processes is 90–400 *μ*m; cells cannot migrate, if pores are too small and cells cannot attach, if there is not enough surface area [13, 14]. Therefore, the foam with a polyurethane film dressing displayed an appropriate structure for the wound to heal. The multilayer structure of hydrocolloid with a foam layer dressing and foam with a hydrogel and polyurethane film layer dressing were clearly identified. Both alginate dressings showed a fibrous structure. The contrasting structures with or without using different components within the wound dressing affected the various properties.

### 3.2. Absorption Properties

**Figure 2:**
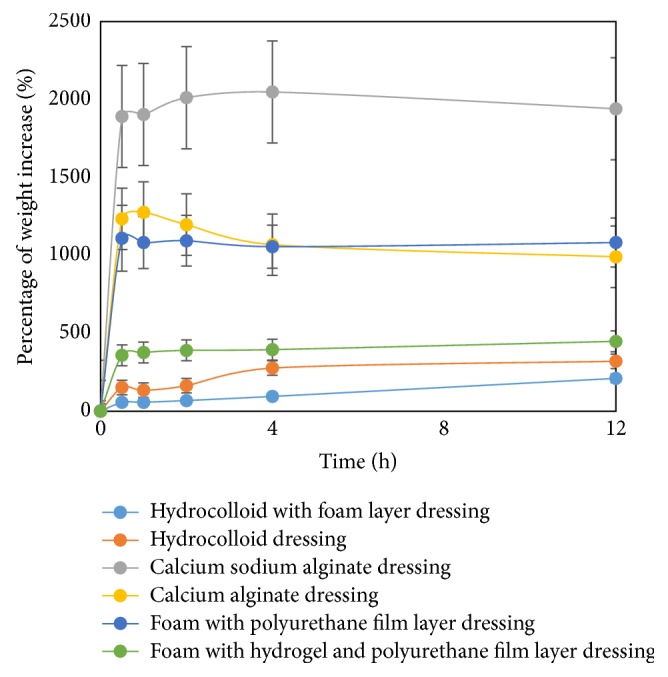
Absorption properties.

This was the first study to demonstrate the absorption characteristics of a wound dressing during a 12-hour period. The absorption properties of the wound dressing are shown in Figure 2. The calcium sodium alginate dressing had the highest absorption capacity and after 4 hours, it degraded. However, the calcium alginate dressing degraded after 1 hour due to the difference in the chemical structure. The sodium ions in an alginate dressing stimulate the gel formation resulting in a high absorption capacity [15]. The calcium sodium alginate dressing showed a slow degradation probably because of the high glucuronic acid content [6, 15]. The researchers found that the number of pores was an important factor affecting the absorption capacity among the foam dressings (Figure 1). The absorption capacity of foam with a polyurethane film layer dressing was higher when compared to other foam dressings and was almost equivalent to a calcium alginate dressing. Both of the hydrocolloid dressings had a low absorption capacity and absorbed exudates slowly. Interestingly, the absorption capacity of hydrocolloid with a foam layer dressing was lower when compared with the hydrocolloid dressings. This indicated that the multilayer structure was not usually associated with increasing absorption properties.

### 3.3. Dehydration Properties

**Figure 3:**
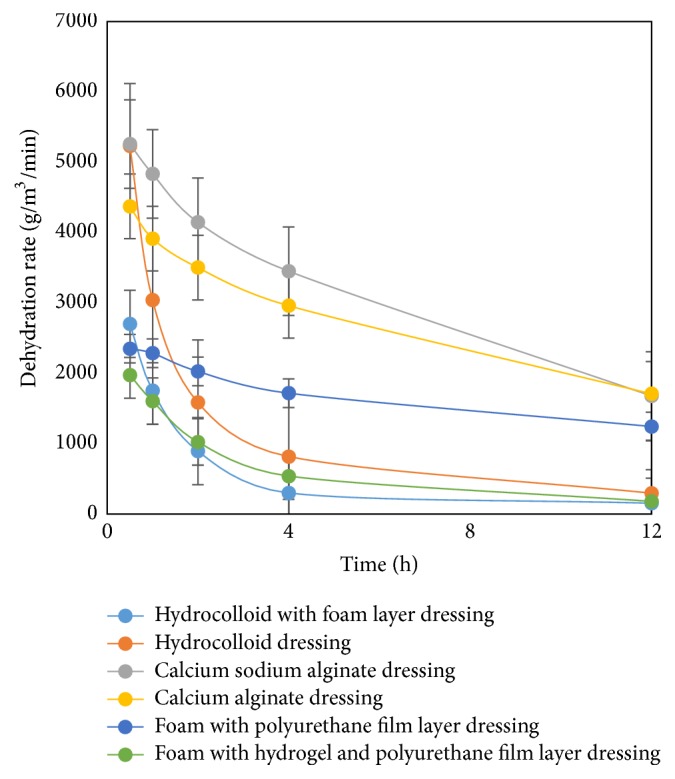
Dehydration properties.

The dehydration properties of the wound dressings are shown in Figure 3. The alginate dressings had the highest dehydration rate. The foam with a polyurethane film layer dressing demonstrated a moderate rate of dehydration while the dehydration rate of foam with a hydrogel and polyurethane film layer dressing was lower when compared to foam with a polyurethane film layer dressing. This may cause maceration because it has a low-to-moderate absorption capacity, which would interfere with the wound's healing process [7]. The wound dressing should have a balance between the absorption capacity and dehydration rate to prevent maceration. The hydrocolloid dressing had a high dehydration rate in the first 30 minutes, then it decreased dramatically. Hydrocolloid with a foam layer dressing also had a low dehydration rate. However, both of the hydrocolloid dressings absorbed exudate slowly resulting in nonmaceration.

### 3.4. Water Vapour Transmission Rate

**Figure 4:**
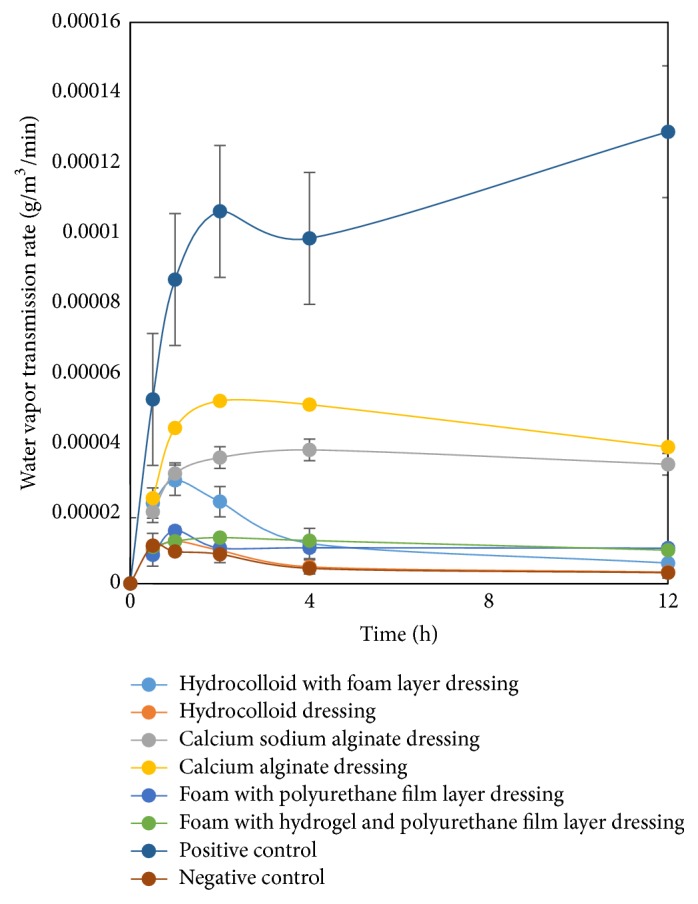
Water vapour transmission rate.

The water vapour transmission rate (WVTR) of the wound dressings is shown in Figure 4. One of the important properties of an ideal wound dressing would allow permeable water vapour to avoid the accumulation of the wound exudates [5, 6]. These results showed that the hydrocolloid dressings could not allow for permeable water vapour whereas other dressings showed the appropriate properties, especially the alginate dressings. Therefore, if an innovative hydrocolloid dressing is to be developed with these properties, it would require creating a multilayer structure.

### 3.5. Dispersion Characteristics

**Figure 5:**
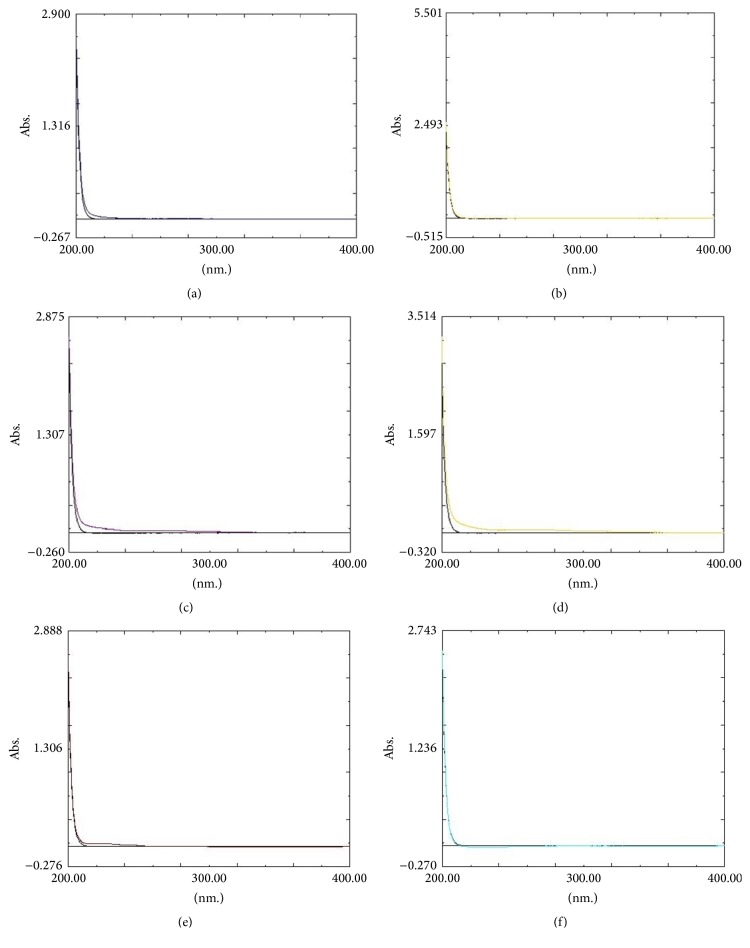
Dispersion characteristics compared with a pseudo-wound exudate: (a) hydrocolloid with a foam layer dressing, (b) hydrocolloid dressing, (c) calcium sodium alginate dressing, (d) calcium alginate dressing, (e) foam with a polyurethane film layer dressing, and (f) foam with a hydrogel and polyurethane film layer dressing.

The dispersion characteristics of the wound dressings are shown in Figure 5. The dispersions were shown visually from both alginate dressings resulting in the difficulty of removing the dressing. In addition, the spectra of the pseudo-wound exudate after being submerged with the alginate dressings were not quite similar to those of the pseudo-wound exudate, but other wound dressings were not dispersed in a pseudo-wound exudate. The wound dressing should retain the original structure after being submerged with a wound exudate for painless removal.

## 4. Conclusions

Calcium sodium alginate dressings demonstrate the highest quality among absorbent wound dressings. However, the integrity of alginate dressings should be taken into consideration and further research should be undertaken.

## Abbreviations

WVTR: Water vapour transmission rate
SEM: Scanning electron microscope.
